# Ultrasound-Guided Combined Interscalene-Cervical Plexus Block for Surgical Anesthesia in Clavicular Fractures: A Retrospective Observational Study

**DOI:** 10.1155/2018/7842128

**Published:** 2018-06-03

**Authors:** Onur Balaban, Turan Cihan Dülgeroğlu, Tayfun Aydın

**Affiliations:** ^1^Department of Anesthesiology and Pain Medicine, School of Medicine, Dumlupinar University, Kutahya, Turkey; ^2^Department of Orthopedic and Trauma Surgery, School of Medicine, Dumlupinar University, Kutahya, Turkey

## Abstract

**Objective:**

We aim to report our experiences regarding the implementation of the ultrasound-guided combined interscalene-cervical plexus block (CISCB) technique as a sole anesthesia method in clavicular fracture repair surgery.

**Materials and Methods:**

Charts of patients, who underwent clavicular fracture surgery through this technique, were reviewed retrospectively. We used an in-plane ultrasound-guided single-insertion, double-injection combined interscalene-cervical plexus block technique. During the performance of each block, the block areas were visualized by using a linear transducer, and the needles were advanced by using the in-plane technique. Block success and complication rates were evaluated.

**Results and Discussion:**

12 patients underwent clavicular fracture surgery. Surgical regional anesthesia was achieved in 100% of blocks. None of the patients necessitated conversion to general anesthesia during surgery. There were no occurrences of acute complications.

**Conclusions:**

The ultrasound-guided combined interscalene-cervical plexus block was a successful and effective regional anesthesia method in clavicular fracture repair. Prospective comparative studies would report the superiority of the regional technique over general anesthesia.

## 1. Introduction

Clavicle fractures account for 35% of injuries to the shoulder girdle and generally occur after blunt traumas. For displaced clavicle fractures with greater than 2 cm of shortening, current recommendation is operative management with open reduction and internal fixation [1, 2].

Clavicle surgery is usually performed under general anesthesia. Any regional anesthesia method for repair of a clavicular fracture has not been described and not commonly performed in current anesthesia practice. Although peripheral nerve blocks are commonly used for a wide variety of surgical procedures on the upper extremity, there are very few reports regarding regional anesthesia for surgery of the clavicle. In the literature, proposed interventional strategies for clavicular fractures include superficial cervical plexus blocks, combined cervical plexus-deep cervical plexus blocks, and interscalene brachial plexus blocks. These techniques are usually used for analgesia of the clavicle [1]. Choosing the optimal nerve block to anesthetize the clavicle requires a thorough understanding of innervation, which remains controversial. The sensory innervation of the clavicle has been attributed to either the cervical or brachial plexus [3, 4].

Ultrasound-guided techniques have enabled the anesthetists to reduce doses of local anesthetic drugs and perform more successful blocks [5, 6]. As local anesthetic doses were reduced with the use of USG and lower doses were administered, combined or multiple blocks have become possible.

In regard to the neuronal anatomy and clinical experience, the combined interscalene-cervical plexus block seems to be an effective block and may be a promising method for sufficient surgical anesthesia in clavicle surgery. We understand from very few case reports that interscalene brachial plexus blocks and combined interscalene-cervical plexus blocks are being used as a single anesthetic modality for surgery of the clavicle in some hospitals [7]. Up to date, there is neither a prospective study nor a well-established regional anesthesia method for clavicle surgery.

The objective of our retrospective analysis is to demonstrate that combined interscalene-cervical plexus block is effective and safe as a sole anesthesia method for the patients undergoing clavicle fracture repair. We present a case series of clavicular fractures that were operated under combined interscalene-cervical plexus blocks.

## 2. Materials and Methods

Patient charts were retrospectively reviewed starting from May 2014. All patients were informed about the treatment, surgery, and anesthesia method before the procedure. Informed consents for the surgery and anesthesia method were obtained. Block success, acute complications as inadvertent arterial puncture, hematoma formation, respiratory distress, Horner's syndrome, pneumothorax, and signs of local anesthetic toxicity were evaluated.

### 2.1. Anesthesia Method

**Figure 1:**
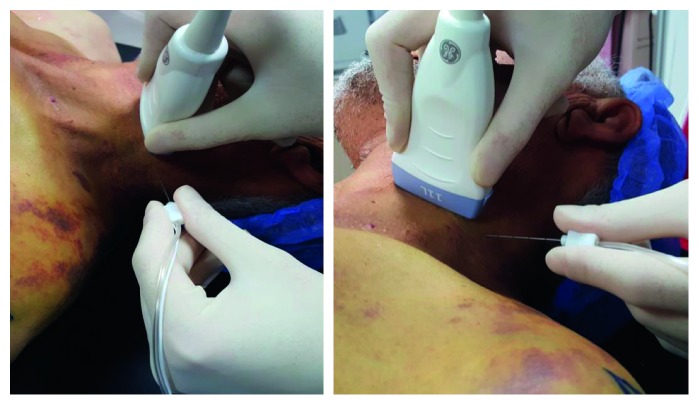
Position of the ultrasound transducer and the needle during the performance of blocks.

**Figure 2:**
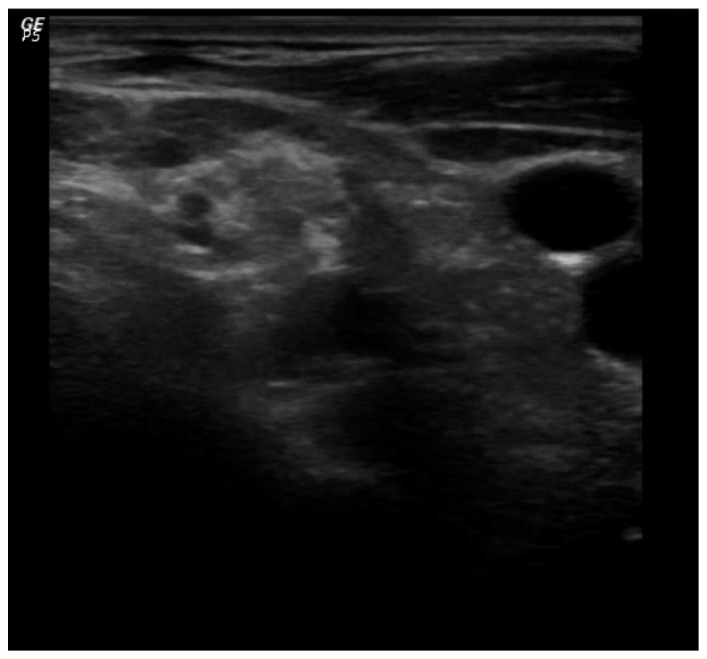
Visualization of the anatomical structures at the midneck level in the transverse plane: sternocleidomastoid muscle, carotid artery, and jugular vein. Brachial plexus can be seen as three hypoechoic nodular structures between the scalene muscles.

We define this technique as the in-plane ultrasound-guided single-insertion, double-injection combined interscalene-intermediate cervical plexus block. Cervical plexus blocks combined with interscalene blocks were performed under ultrasound guidance (LOGIQ P5, GE Healthcare, Milwaukee, WI, USA). The skin was prepared using an antiseptic solution, and the transducer was dressed with a sterile cover. A 12-megahertz linear transducer (GE Healthcare, Milwaukee, WI, USA) was used for performing the blocks. The related side of the patient was scanned by ultrasound in a transverse orientation across the neck with the probe marker facing lateral at the level of the interscalene groove (Figure 1). A long axis view of the brachial plexus roots, sternocleidomastoid (SCM) muscle, levator scapula muscle, carotid artery, jugular vein, and anterior and middle scalene muscles was identified (Figure 2).

**Figure 3:**
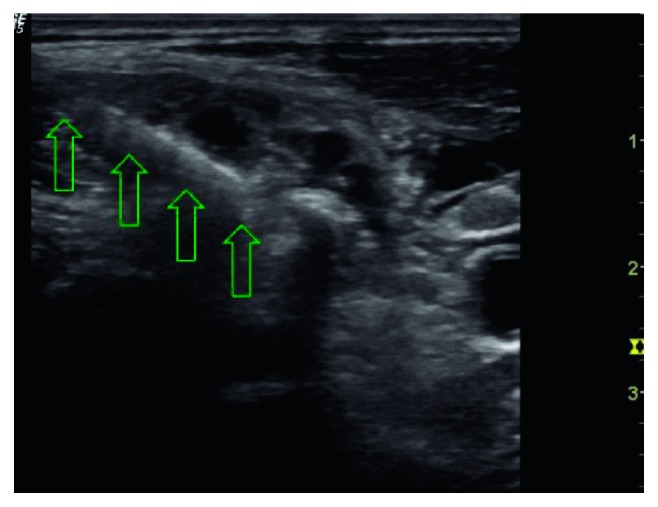
Position of the needle and local anesthetic distribution between the scalene muscles around the nerves in the interscalene region during the performance of the interscalene block. The arrows are showing the body and the tip of the needle.

**Figure 4:**
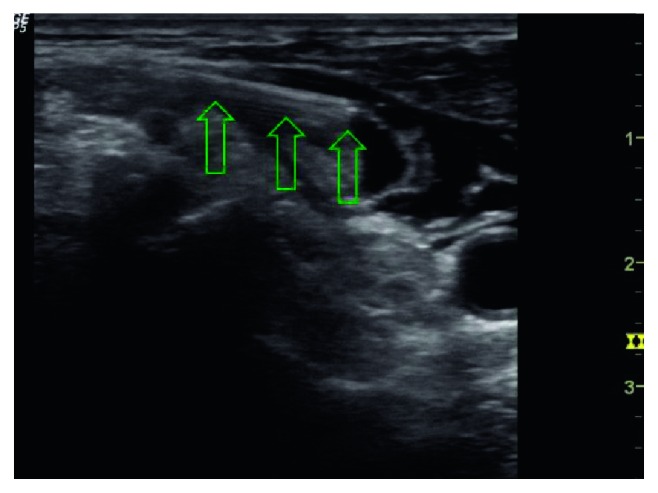
Position of the needle and local anesthetic distribution posterior to the sternocleidomastoid muscle during the performance of the cervical plexus block. The arrows are showing the body and the tip of the needle.

The blocks were performed using a 5-centimeter block needle (Stimuplex Ultra, Braun, Melsungen, Germany). Initially, an interscalene block was performed under US guidance. The US transducer was placed transversally on the neck at the level of the superior pole of the thyroid cartilage and then slightly aligned laterally where the nerve roots were observed between the anterior and middle scalene muscles at the interscalene groove. The needle insertion site was determined under US guidance as the point where the posterolateral border of the SCM muscle starts. The final target position of the needle was immediately posterior to the space between the C5 and C6 roots (Figure 3). The needle was inserted laterally at the posterior border of the SCM muscle and advanced under US guidance using the in-plane technique. 0.5 ml/kg of the local anesthetic drug (0.5% bupivacaine) was administered under real-time visualization of local anesthetic distribution. After the performance of the interscalene block, the needle was withdrawn and redirected to the cervical plexus. The cervical plexus block was performed as a plane block in the prevertebral fascia posterior to the SCM muscle. The hyperechoic fascia of the SCM muscle on its posterolateral border was identified, and the needle was advanced along the posterior border of the SCM muscle under real-time US guidance to the nerve point of the neck. The needle tip was positioned to inject local anesthetic deep to the SCM muscle along its tapering posterolateral border but superficial to the prevertebral fascia (Figure 4).

At this level, visualization of the nerves can be difficult and cannot sometimes be identified, and it is not necessary to determine nerve structures in the fascial plane. We proceeded to inject to fill the posterior plane of the SCM muscle, where the cervical nerves exist to achieve an effective cervical plexus block and surgical anesthesia. Distribution of the local anesthetic drug was visualized during the procedure (Figures 3 and 4). Half of the total volume of the local anesthetic drug was administered for interscalene blocks, and the remaining half was administered for cervical plexus blocks. Motor blockade was determined by loss of shoulder abduction, and sensory blockade was assessed using the pinprick test at the surgery site. The patient was also checked for pain with mobilization of the arm and palpation of the clavicle by the surgeon. A successful block was defined as one which did not necessitate conversion to general anesthesia. Postoperative analgesia was achieved by intravenous tramadol when necessary. As this procedure also affects the phrenic nerve, it was not performed on those with coexisting cardiac or respiratory disease.

Descriptive statistics of the study are calculated, and the data were analyzed using SPSS Statistics 21.0 program (IBM Corporation, NY, USA). Continuous quantitative data were expressed as number, mean, and standard deviation, and qualitative data were expressed as number and percentage.

## 3. Results

**Figure 5:**
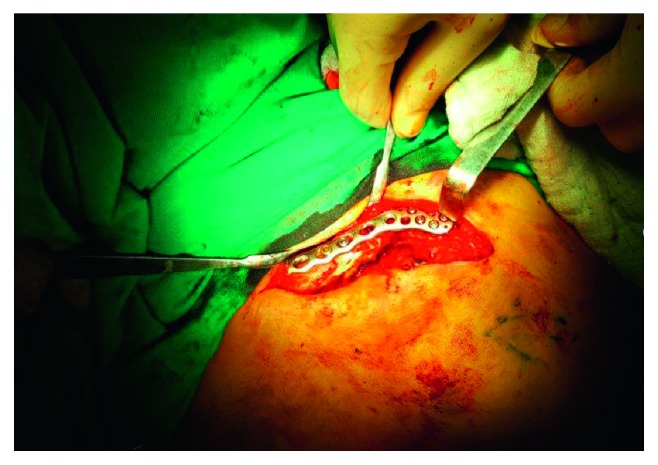
The operation site and clavicular fixation.

The patient characteristics are summarized in Table 1. Totally, 12 patients underwent clavicle operation. Eleven patients underwent open reduction and internal fixation of the clavicle fracture (Figure 5). One patient underwent removal of the implant from the clavicle. One of the patients had liver disease, and one patient had diabetes mellitus. Other patients' previous medical history was unremarkable.

The patients were transported to the operating room where standard monitors (electrocardiograph, noninvasive blood pressure, and pulse oximetry) were applied. All blocks were performed in the operating room. Resuscitative measures were present during the performance of blocks and during the operation. All surgeries were performed in supine position.

All patients completed their operations under regional anesthesia, and no patient required conversion to general anesthesia. One of the patients complained of mild pain at the initiation of the surgery. One other patient felt pain with manipulation of the clavicle and required a deeper sedation. 50 micrograms of fentanyl and 50 milligrams of ketamine were administered intravenously to the patients, and the operations continued uneventfully. There was no need of anticholinergic drugs as no side effect of ketamine was observed. We considered these patients as successful because both patients did not need to be intubated and continued to have effective respiration in the remaining course. We did not detect a significant change in blood pressures and heart rates intraoperatively. No surgical complications and early complications related to the blocks occurred. None of the patients developed Horner's syndrome. Outcomes of surgery and anesthesia are summarized in Table 2.

We asked the surgeons about their satisfaction about the anesthesia method. The surgeons' satisfaction was good, and none of them declared negative opinion about the anesthesia method. They were in favor of this method, which may be useful especially for high-risk patients.

## 4. Discussion

This case series demonstrated that a combined interscalene-intermediate cervical plexus block under ultrasound guidance is feasible in clavicular fracture surgery. Before ultrasound, local anesthetic doses required for successful blocks were substantially high; therefore, the risk for systemic local anesthetic toxicity was high. Advances in the field of ultrasound-guided peripheral nerve blocks have allowed reduction of local anesthetic doses in interscalene blocks [8]. Ultrasound-guided interscalene blocks are performed commonly in our clinic for shoulder surgeries. Cervical plexus blocks are also performed under ultrasound guidance for endarterectomy operations. The idea of using a combination of two blocks was encouraged by and came up after reduction of local anesthetic doses we used to administer to 10–20 milliliters. In consultation with the trauma surgeons, with the guarantee of converting to general anesthesia if surgical pain is felt, we have been offering this method to our patients undergoing clavicular surgery as an alternative to general anesthesia since 2014.

Understanding cervical plexus anatomy, innervation of the clavicle and innervation of the skin over the surgical site is important to establish a regional anesthesia method for clavicular surgery. The ventral rami of the first four cervical spinal nerves constitute the cervical plexus. They are located in front of the C1 to C4 vertebra, deep and posterior to the sternocleidomastoid (SCM) muscle. The plexus gives 4 terminal branches: greater auricular, lesser occipital, supraclavicular, and transverse cervical nerves. They provide sensory innervation to the skin and superficial structures of the anterolateral neck and sections of the ear and shoulder. The branches emerge at the posterior border of the sternocleidomastoid muscle, anterolateral to the levator scapulae and middle scalene muscles at the level of the superior pole of the thyroid cartilage [9, 10].

These nerves enter the skin at the middle of the posterior border of the sternocleidomastoid muscle at the level of C3, a point which lies superior to the locus and was inappropriately termed as Erb's point [11]. Some authors include the fifth cervical nerve to the plexus which contributes to the formation of one of the motor branches of the cervical plexus called the phrenic nerve. Therefore, the cervical plexus can also be defined as a network of nerves formed by the ventral rami of C1–C5 nerves and gives off both motor and sensory branches [12]. The sensory innervation of the clavicle and the overlying skin is not clearly identified and varies depending on the source in the literature between C3 and C6. The supraclavicular, subclavian, and long thoracic/suprascapular nerves, alone or together, may be responsible for pain transmission after clavicular fracture and surgery [1, 13].

Proposed interventional strategies for clavicular fractures include superficial cervical plexus blocks, combined superficial-deep cervical plexus blocks, and interscalene brachial plexus blocks [1]. Cervical plexus blocks are used as a sole anesthesia method in many surgeries such as carotid endarterectomies, dental procedures, submandibular and submental abscess drainage, minimally invasive thyroidectomy, and Zenker's diverticulectomy. Especially in carotid endarterectomies, superficial, intermediate, and deep cervical plexus blocks are widely performed [12, 14, 15]. An ultrasound-guided interscalene block is also a well accepted method in anesthesia practice which is preferred to achieve surgical anesthesia in shoulder surgeries such as arthroscopy, rotator cuff repair, and reductions of shoulder joint dislocations.

Nevertheless, general anesthesia seems to be extensively preferred in clavicular surgery in anesthesia practice. Fear of block failure has been overcome with regularly used regional anesthesia, and an improved block success rate is accomplished with the use of ultrasound-guided blocks. Thereafter, we could be able to change the standard of daily institutional practice of clavicle surgeries performed under general anesthesia to regional anesthesia.

For performance of ultrasound-guided cervical plexus blocks, the aim is to place the needle tip underneath the plexus if visualized. If the plexus is not visualized easily, the needle tip should be placed deep under the SCM muscle, in the plane of the prevertebral fascia [12]. An anatomical study suggests the compartment between the superficial layer and the prevertebral layer of the cervical fascia as a suitable target for cervical plexus blocks. This injection site describes an intermediate cervical plexus block [16]. Anatomically, with superficial blocks, there could be spread of the injectate to structures beneath the deep cervical fascia. This was also observed with real-time US in our study. The superficial cervical space communicates with the deep cervical space, and this may explain the efficacy of the superficial cervical plexus blocks [17].

There is a confusing nomenclature in the articles about cervical plexus blocks. Existing literature indicates that there have been various methods described for the proper injection technique in superficial cervical plexus blocks. The classical technique of superficial cervical plexus blocks was described as subcutaneous injection of the local anesthetic drug, which was found clinically effective for carotid endarterectomy [18]. In some reports, superficial cervical plexus injections have been suggested to be “intradermal” (even more superficial) or to be administered into the body of the sternocleidomastoid muscle. The subinvesting fascia injection might be termed as the “intermediate cervical plexus block” [19]. We preferred the term “intermediate cervical plexus block” which would describe our method correctly as local anesthetic distribution was within the prevertebral fascia in our study.

Ultrasound-guided superficial cervical plexus blocks are found to be successful for treating pain in emergency care settings [9]. Superficial cervical plexus blocks can also be used to provide surgical anesthesia for lymph node biopsy and excision of a thyroid nodule and placement of hemodialysis catheters [20, 21]. Ultrasound-guided bilateral cervical plexus blocks could be performed for postoperative analgesia following thyroid surgeries [22]. In oral and maxillofacial surgical practice and in selected neck surgeries, use of superficial cervical plexus blocks was offered as an alternative to general anesthesia [23, 24].

The combined interscalene-cervical plexus block is a novel method, which was reported in very few cases. Vandepitte et al. used this technique successfully as a primary anesthesia method in a pregnant patient who had a clavicular fracture [7]. They found this method effective for achieving surgical anesthesia. Shanthanna reported two cases of clavicular surgery operated under general anesthesia [25]. The patients were performed superficial cervical plexus block and selective C5 nerve root block under ultrasound guidance, along with general anesthesia. Both patients had an effective regional block and required minimal supplementation of analgesia, both being discharged on the same day.

## 5. Limitations of the Study

This clinical series is limited by its retrospective nature, and patients were not followed for a postoperative analgesia requirement. This may be a subject of prospective study in the future. As clavicular repair is a rarely performed intervention, the low number of cases was also a limitation. Several measurements were not evaluated such as the number of needle insertion attempts, needle redirections, block performing times, and onset times. Long-term complications were also not evaluated.

## 6. Conclusions

Our limited experience suggests that the combined interscalene-cervical plexus block is possible as a sole anesthesia method in patients who undergo clavicular fracture surgery. In this case series, regional anesthesia was successful, effective, and well tolerated in all of the patients. This method may be considered as an alternative to general anesthesia. Prospective (randomized) trials are required to determine which constitutes the best option for such operations.

## Figures and Tables

**Table 1:** Patient characteristics.

	Minimum	Maximum	Mean ± standard deviation
Age (years)	15.00	70.00	34.33 ± 20.11
Height (m)	1.67	1.87	1.74 ± 0.07
Weight (kg)	56.00	85.00	72.33 ± 10.63
BMI (kg/m^2^)	17.72	28.73	24.11 ± 4.58

**Table 2:** Outcomes of surgery and anesthesia.

Surgery duration (minutes) (mean ± standard deviation)	73.75 ± 17.02
Acute complications	None
Block success rate (%)	100
